# A bioflocculant from *Corynebacterium glutamicum* and its application in acid mine wastewater treatment

**DOI:** 10.3389/fbioe.2023.1136473

**Published:** 2023-02-28

**Authors:** Yinlu Liu, Yan Zeng, Jiangang Yang, Peng Chen, Yuanxia Sun, Min Wang, Yanhe Ma

**Affiliations:** ^1^ Tianjin Key Laboratory of Industrial Microbiology, College of Biotechnology, Tianjin University of Science and Technology, Tianjin, China; ^2^ National Engineering Laboratory for Industrial Enzymes, Tianjin Institute of Industrial Biotechnology, Chinese Academy of Sciences, Tianjin, China

**Keywords:** bioflocculant, *Corynebacterium glutamicum*, structural characterization, flocculating activity, acid mine wastewater

## Abstract

Although many microorganisms have been found to produce bioflocculants, and bioflocculants have been considered as attractive alternatives to chemical flocculants in wastewater treatment, there are few reports on bioflocculants from the safe strain *C. glutamicum*, and the application of bioflocculants in acid wastewater treatment is also rare attributed to the high content of metal ions and high acidity of the water. In this study, a novel bioflocculant produced by *Corynebacterium glutamicum* Cg1-P30 was investigated. An optimal production of this bioflocculant with a yield of 0.52 g/L was achieved by Box–Behnken design, using 12.20 g/L glucose, 4.00 g/L corn steep liquor and 3.60 g/L urea as carbon and nitrogen source. The structural characterization revealed that the bioflocculant was mainly composed of 37.50% neutral sugar, 10.03% uronic acid, 6.32% aminosugar and 16.51% protein. Carboxyl, amine and hydroxyl groups were the functional groups in flocculation. The biofocculant was thermally stable and dependent on metal ions and acidic pH, showing a good flocculating activity of 91.92% at the dosage of 25 mg/L by aid of 1.0 mM Fe^3+^ at pH 2.0. Due to these unique properties, the bioflocculant could efficiently remove metal ions such as Fe, Al, Zn, and Pb from the real acid mine wastewater sample without pH adjustment, and meanwhile made the acid mine wastewater solution become clear with an increased neutral pH. These findings suggested the great potential application of the non-toxic bioflocculant from *C. glutamicum* Cg1-P30 in acid mine wastewater treatment.

## Introduction

The removal of colloidal particles mediated by flocculants is an important procedure in many industrial fields such as tap water purification and wastewater treatment, dyes and textiles, food and beverage. Flocculants are generally divided into three different categories: inorganic, organic, and bioflocculants (Alias et al., 2022). Compared with conventional chemical inorganic and organic flocculants, microbial bioflocculants, the macromolecules secreted by microorganisms during growth and lysis, are considered to be ecosystem-friendly due to their non-toxic and biodegradable properties. In addition, microbial bioflocculants can be produced at high rates and the extracellular bioflocculants are easily recovered from the fermentation broth. Thus, microbial biofluccolants are attracting extensive attention as a potential alternative to chemical flocculants (Shahadat et al., 2017).

A number of microorganisms, including bacteria, fungi, microalgae and actinomycetes, have been reported to produce bioflocculants. For example, *Bacillus* sp. is the most studied bacterial species for bioflocculant production (Bakar et al., 2021). However, the practical application of bioflocculants is still limited due to their low flocculating efficiency, low yield, and high cost of production. Consequently, it become important to screen novel effective bioflocculant-producing microorganisms, seek for economical cultivation substrates and optimize the fermentation process in the research of bioflocculant (Okaiyeto et al., 2016). At the same time, the performance of biological flocculants is closely related to its structural composition (Kurniawan et al., 2022), and is greatly affected by flocculation conditions such as dosage, metal ions, pH value, and temperature. Therefore, it is beneficial to master this information for selecting suitable bioflocculants facing with application scenarios with different characteristics. *Corynebacterium glutamicum* is generally recognized as safe (GRAS) and have been widely used as host for production of functional compounds, however, to date there have been few reports on production, characterization, properties of biofluccolants from *Corynebacterium glutamicum*, except a polygalacturonic acid bioflocculant named REA-11 from *C. glutamicum* CCTCC M201005 (He et al., 2002; Li et al., 2003; He et al., 2004a; He et al., 2004b).

Acid mine wastewater, which is an unavoidable by-product of the mining and mineral industry, is harmful to the aquatic environment because it contains high concentrations of dissolved heavy metals (primarily iron, aluminum, zinc, and other heavy metals) and sulphate, and have a high turbidity and a high acidity with a pH of 2.0–3.0 (Feng et al., 2004; Kefeni et al., 2017; Fu et al., 2020). Acid mine wastewater has become the leading environmental issue of the mining industry over the years, many methods, containing alkaline chemical neutralization, precipitation, adsorption, and membrane technology have been widely used in treatment of acid mine wastewater. However, though the utilization of biolflocculant is thought as an economic and effective strategy to treat the mining wastewater, such as clarification of turbid aqueous solutions (Liu et al., 2019), removal of organics (Maliehe et al., 2019) and metal remediation (Ayangbenro et al., 2019), probably because the flocculating activities of many reported bioflocculants have been significantly inhibited in high acidic conditions or in the presence of iron or aluminum (Tawila et al., 2019; Pu et al., 2020; Rajivgandhi et al., 2021), the application of bioflocculant in acid mine wastewater treatment is still need to be explored.

In this study, a novel bioflocculant was produced from *C. glutamicum* Cg1-P30, which was Gram-positive, catalase positive with rod-like shapes and identified by 16S rRNA sequence analysis (Supplementary Material), and the culture medium using low-cost corn steep liquor as a nutrient source for fermentation was optimized. The structural characterization, flocculating activities, and the application in treatment of acid mine wastewater of the isolated bioflocculant were also investigated.

## Materials and methods

### Culture conditions

*Corynebacterium glutamicum* Cg1-P30 (CMCC NO. 22680), preserved at China General Microbiological Culture Collection Center, was initially grown in brain heart infusion (BHI) agar medium for activation. The activated strain was maintained on BHI broth at 30°C in a rotary shaker at 200 rpm overnight. Subsequently, 2% of the seed culture was transferred into new flasks containing 50 mL of production medium consisting of 10 g/L glucose, 5 g/L corn steep liquor, 5 g/L urea, 0.25 g/L MgSO_4_·7 H_2_O, 1.0 g/L K_2_HPO_4_ and 1.0 g/L KH_2_PO_4_ and incubated at 30°C and 200 rpm for 48 h. The concentrations of glucose, corn steep liquor, and urea were further optimized as described in the section of optimization of medium for bioflocculant production.

### Optimization of medium for bioflocculant production

Based on the result of one-factor-at-a-time experiment, response surface methodology through Box-Behnken design (Design Expert software, Version 8.0.6) was applied to evaluate the optimum level and interactive effects of carbon and nitrogen sources on bioflocculant production. The concentration of glucose, corn steep liquor, and urea were selected as independent variables at three different levels, while the response variable was the flocculating activity of the fermentation supernatant after culturing for 48 h. The result of Box-Behnken design was shown in Table 1. A quadratic polynomial equation was fitted to evaluate the correlate relationship between the independent variables and the response as following:

$$Y=\beta_{0}+\beta_{1}X_{1}+\beta_{2}X_{2}+\beta_{3}X_{3}+\beta_{12}X_{1}X_{2}+\beta_{13}X_{1}X_{3}+\beta_{23}\;X_{2}X_{3}+\beta_{11}\;X_{1}^{2}+\beta_{22}\;X_{2}^{2}+\beta_{33}\;X_{3}^{2}$$

Where Y (%) is the flocculating activity of the fermentation supernatant, β_0_ is the intercept coefficient; β_1_, β_2_, and β_3_ are the linear coefficients; β_11_, β_22_, and β_33_ are the quadratic coefficients; and β_12_, β_13_, and β_23_ are the interaction coefficients; X_1_, X_2_, X_3_ are the concentration of glucose, corn steep liquor, and urea, respectively. Three additional experiments were conducted to verify the validity of the statistical experimental strategies.

**TABLE 1:** The matrix of the BBD experiment for culture medium optimization and the experimental and predicted flocculating activity.

Run	X_1_	X_2_	X_3_	Flocculating activity (%)	
	(Glucose)	(Corn steep liquor)	(Urea)		
	Real level (g/L)	Real level (g/L)	Real level (g/L)	Predicted measured	Measured
1	10	3	4	76.02	76.13 ± 0.81
2	15	3	4	72.89	71.67 ± 0.80
3	10	5	4	73.53	74.74 ± 0.23
4	15	5	4	68.70	68.59 ± 0.71
5	10	4	3	81.50	80.89 ± 0.70
6	15	4	3	75.78	76.50 ± 1.11
7	10	4	5	68.86	68.15 ± 1.00
8	15	4	5	66.64	67.24 ± 0.56
9	12.5	3	3	82.97	83.46 ± 1.13
10	12.5	5	3	77.57	76.95 ± 0.64
11	12.5	3	5	70.02	70.63 ± 0.60
12	12.5	5	5	68.75	68.26 ± 0.57
13	12.5	4	4	87.49	86.37 ± 0.61
14	12.5	4	4	87.49	87.50 ± 0.61
15	12.5	4	4	87.49	88.04 ± 0.66
16	12.5	4	4	87.49	87.97 ± 0.43
17	12.5	4	4	87.49	87.54 ± 0.94

### Determination of flocculating activity

The flocculating activity was determined using the kaolin clay suspension method (Sun et al., 2012) with some modification. 1 mL bioflocculant sample (the fermentation supernatant was used in the optimization experiment) and 50 μL FeCl_3_ (1 M) solution were mixed with 49 mL of kaolin solution (4 g/L) and then incubated for 5 min at room temperature. The absorbance of the upper phase of the mixture was measured by at 550 nm. A control in which the bioflocculant was replaced with deionized water was also conducted under the same conditions. The flocculating activity was calculated according to the following equation:

$$Flocculating\;activity=\left[\left(B-A\right)/B\right]\times 100\%$$

Where A and B are the absorbance at 550 nm of the sample and control, respectively.

### Extraction and purification of bioflocculant

The cell-free supernatant obtained from the culture broth by centrifugation at 6,000 rpm and 4°C for 30 min was mixed with three volumes of ethanol and kept at 4°C overnight for precipitation. The resulting precipitate by centrifugation was dissolved in distilled water to repeat the precipitation procedure for three times. The final collected precipitate was dissolved in deionized water, dialyzed (molecular weight cutoff of 10,000 Da) to remove low molecular weight compounds and lyophilized at −50°C to obtain the purified bioflocculant.

### Characterization of the bioflocculant

The total content of polysaccharide, uronic acid, amino sugar and protein in the bioflocculant was measured by the phenol-sulfuric acid method, carbazole-sulfate method, Elson-Morgan method and Bradford method, with glucose, glucuronic acid, glucosamine and bovine albumin as the standard, respectively. For monosaccharide composition analysis, after hydrolysis of the bioflocculant with trifluoroacetic acid at 110°C for 6 h, the hydrolyzed sample was analyzed by using a high-pressure ion chromatography system (Dionex ICS 3000, Thermo Fisher Scientific Inc., United States), equipped with an anion exchange column (Dionex CarboPac PA20, Thermo Fisher Scientific Inc., United States) and a pulsed amperometric detector (reference electrode Ag-AgCl, measuring electrode Au). For neutral monosaccharide analysis, the mobile phase consisting of A (100 mM NaOH) and B (10 mM NaOH), was programmed as follows: 0–5 min, 100% A; 5–25 min, 10% A. For uronic acid monosaccharide analysis, the mobile phase consisting of A (10 mM NaOH) and B (100 mM NaOH containing 1 M sodium acetate), was programmed as follows: 0–20 min, 95% A; 20–30 min, 70% A; 30–35 min, 100% A. The identification of each monosaccharide peak was determined on the basis of the elution time by comparison with standard solutions of different monosaccharides (fucose, arabinose, rhamnose, galactose, glcosamine, glcose, xylose, galacturonic acid and glucuronic acid). The FT-IR spectra of the bioflocculant was recorded after grinded with KBr powder and pressed into a pellet, by a Bruker Vertex 70 spectrometer (Bruker Optics, United States) at the wavelength range of 400–4,000 cm^−1^. The elemental composition of the bioflocculant was analyzed using a Thermo Scientific K-Alpha XPS system (Thermo Fisher Scientific Inc., United States). The surface morphology of the bioflocculant was observed by SEM (Hitachi SU8010, Japan).

### Measurement of the flocculating activity

The influence of pH was tested in the kaolin clay suspension system with a pH of 2.0–8.0 adjusted by 2 M HCl or 2 M NaOH. The cations including NaCl, KCl, MgCl_2_, CaCl_2_, CuCl_2_, FeCl_2_, FeCl_3_, and AlCl_3_ were used to test the effect of metal ions on the flocculating activity at the final concentration of 1.00 mM. The dose effects of the biopolymer (5–30 mg/L) and Fe^3+^ (0.50–1.50 mM) were studied at pH 2.0. The thermal stability was test at the temperature of 20, 40, 60, 80, and 100°C. The zeta potential of kaolin suspension before and after flocculation was analyzed by a Zetasizer (Zetasizer Pro, Malvern, United Kingdom).

### Application in acid mine wastewater

The acid mine wastewater was sampled from iron ore processing plant of Xiangtan City in Hunan Province, China. In order to analyze the application potential of the bioflocculant in acid mine waste water treatment, the biofluccolant was added into the acid mine wastewater at the final concentration of 25 mg/L without pH adjustment. The suspension was shaken at 200 rpm and 30°C for 30 min. Then the flocculating activity was calculated according to the change of turbidity based on the absorbance at 550 nm and the pH change was record by a pH meter ((FiveEasy Plus pH, Mettler, United States). The supernatant was collected for detecting the concentration of Fe, Al, Mn, Cu, Pb, Zn, Ni and Cd by inductively coupled plasma atomic emission spectroscopy (ICP-AES, Hitachi Limited., Japan). The metal removal rate was calculated as follows:

$$Removal\;rate\;\left(\%\right)=\left(C_{0}-C\right)/C_{0}\times 100$$

where C_0_ and C were the initial and final concentrations of the metal, respectively.

### Statistical analysis

Data are expressed as mean values with standard deviation (±SD) from three independent experiments. Statistical comparisons were performed using analysis of variance (ANOVA), and differences at *p* < 0.05 were considered statistically significant.

## Result

### Optimization of fermentation medium composition

A three-level, three-factor Box-Behnken design was used to optimize the medium composition containing corn steep liquor in shake-flask fermentation. The obtained quadratic regression model representing the flocculating activity as a function of the carbon and nitrogen source was written as follows:

$$Y=-255.46+31.16X_{1}+6.54X_{2}+35.07X_{3}-0.17X_{1}X_{2}+0.35X_{1}X_{3}-1.03\;X_{2}X_{3}-1.31\;X_{1}^{2}-6.54\;X_{2}^{2}-6.12\;X_{3}^{2}$$

where Y was the flocculating activity of the fermentation supernatant, X_1_, X_2_, X_3_ were the concentration of glucose, corn steep liquor, and urea, respectively. The statistical significance of the regression model was assessed by *F*-test and *p*-value. The analysis of variance (ANOVA) for the quadratic model was summarized in Table 2. Similarly to the study of Agunbiade et al. (2022), the model *F*-value of 99.09 indicated that the model was highly statistically significant at *p* < 0.0001. The lack of fit *F*-value and *p*-value was found to be 4.4 and 0.093, indicating that the suitability of the model to predict the variations. The *p*-values showed that X_1_, X_2_, X_3_, X_1_^2^, X_2_^2^, and X_3_^2^ were statistically significant at the 95% confidence level. In contrast, X_1_X_2_, X_1_X_3_, and X_2_X_3_ had no significant influence on the flocculating activity. In addition, the value of the determination coefficient (*R* ^2^), the adjusted determination coefficient (R^2^_adj_) and the predicted determination coefficient determination coefficient (R^2^_pred_) were 0.9922, 0.9811, and 0.9015, respectively, indicating a good correlation between experimental and theoretical results. At the same time, a low value (1.36%) of the coefficient of the variation (C.V.) demonstrated the experimental values were precise and reliable.

**TABLE 2:** Variance analysis (ANOVA) of the response surface quadratic model for culture medium optimizaition.

Source	Sum of squares	Df	Mean square	*F* value	*p*-value
Model	988.54	9	109.84	99.09	<0.0001^a^
X_1_	31.65	1	31.65	28.56	0.0011^a^
X_2_	22.27	1	22.27	20.09	0.0029^a^
X_3_	236.77	1	236.77	213.6	<0.0001^a^
X_1_X_2_	0.72	1	0.72	0.65	0.4471
X_1_X_3_	3.02	1	3.02	2.73	0.1425
X_2_X_3_	4.27	1	4.27	3.85	0.0906
X_1_^2^	280.87	1	280.87	253.39	<0.0001^a^
X_2_^2^	179.92	1	179.92	162.32	<0.0001^a^
X_3_^2^	157.90	1	157.9	142.46	<0.0001^a^
Residual	7.76	7	1.11		
Lack of Fit	5.96	3	1.99	4.40	0.093
Pure Error	1.80	4	0.45		
Cor Total	996.30	16			
R-Squared	0.9922				
Adj R-Squared	0.9811				
Pred R-Squared	0.9015				
Adeq Precision	25.8220				
C.V. %	1.36				

^a^ Model terms are significant.

From the regression model analysis, a maximum flocculating activity of the fermentation broth was estimated as 88.93% under the optimal medium composition with glucose at 12.20 g/L, corn steep liquor at 4.00 g/L and urea at 3.60 g/L. Under this optimal condition, the actual flocculating activity was 88.79% and was very close to the predicted value. By combination of repeated precipitation and dialysis, 0.52 g of purified bioflocculant was finally obtained from 1 L of fermentation broth.

### Characterization of the bioflocculant

**FIGURE 1:**
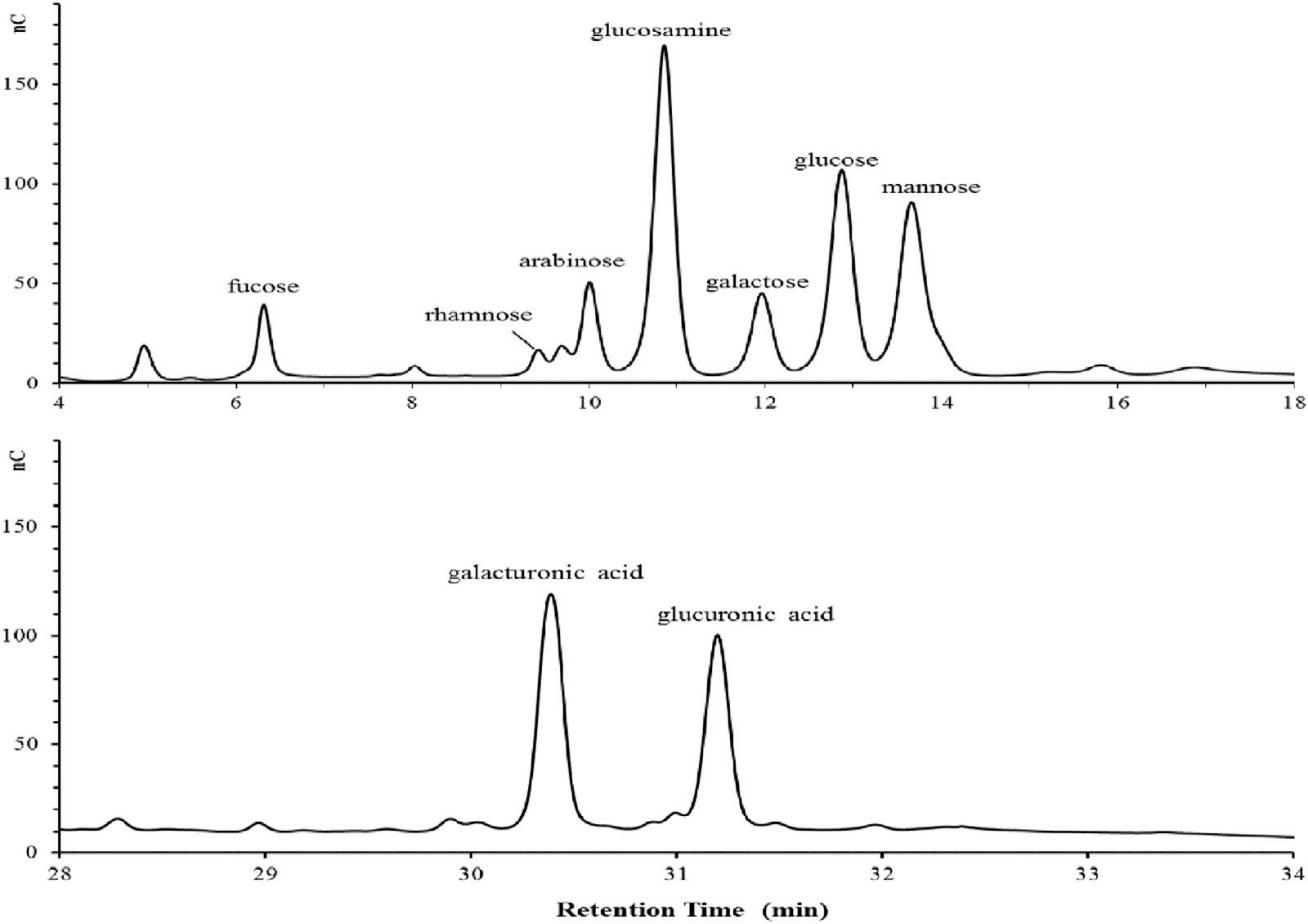
The monosaccharide composition of the bioflocculant detected by ion chromatography.

Chemical analysis revealed that the purified bioflocculant contained neutral sugar (37.50%), uronic acid (10.03%), aminosugar (6.32%), protein (16.51%) and nucleic acid (3.72%). The detected rhamnose, arabinose, galactose, glucose, mannose, fucose, galacturonic acid and glucuronic acid in monosaccharide composition analysis were at a molar ratio of 1.11:2.29:1.84:3.67:4.67:2.93:5.67:4.85 (Figure 1).

**FIGURE 2:**
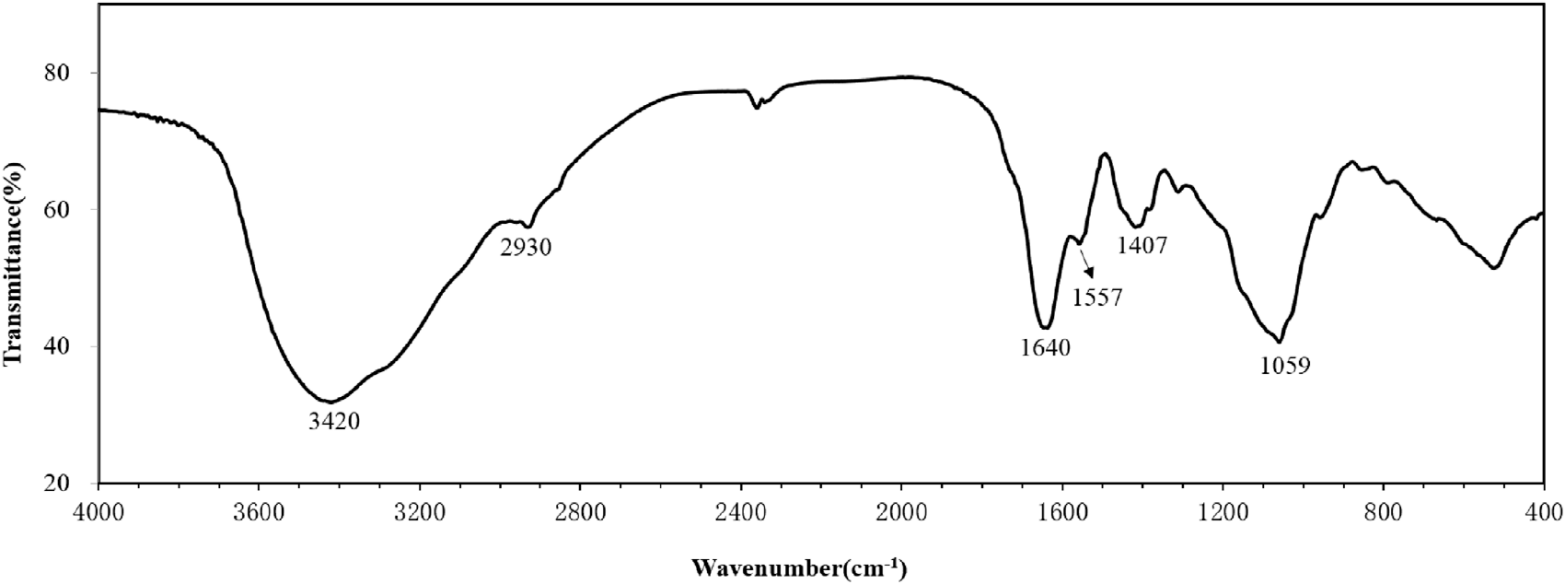
FT-IR spectra for the bioflocculant.

The FTIR spectrum of the purified bioflocculant was displayed in Figure 2. The broad band around 3,420 cm^−1^ was representative of the stretching vibration of O-H group. The weak band at 2,930 cm^−1^ was attributed to the stretching vibration of C-H aliphatic bands (Huang et al., 2019). The band at 1,640 cm^−1^ was resulted from C=O stretching vibration (Poorgholy et al., 2017). The bands at 1,557 and 1,407 cm^−1^ were generated by N-H and C-H bending vibration, respectively (Salaberria et al., 2014; Yang et al., 2022) The band around 1,059 cm^−1^ was from the stretching vibration of the C-O group (Fang et al., 2021).

**FIGURE 3:**
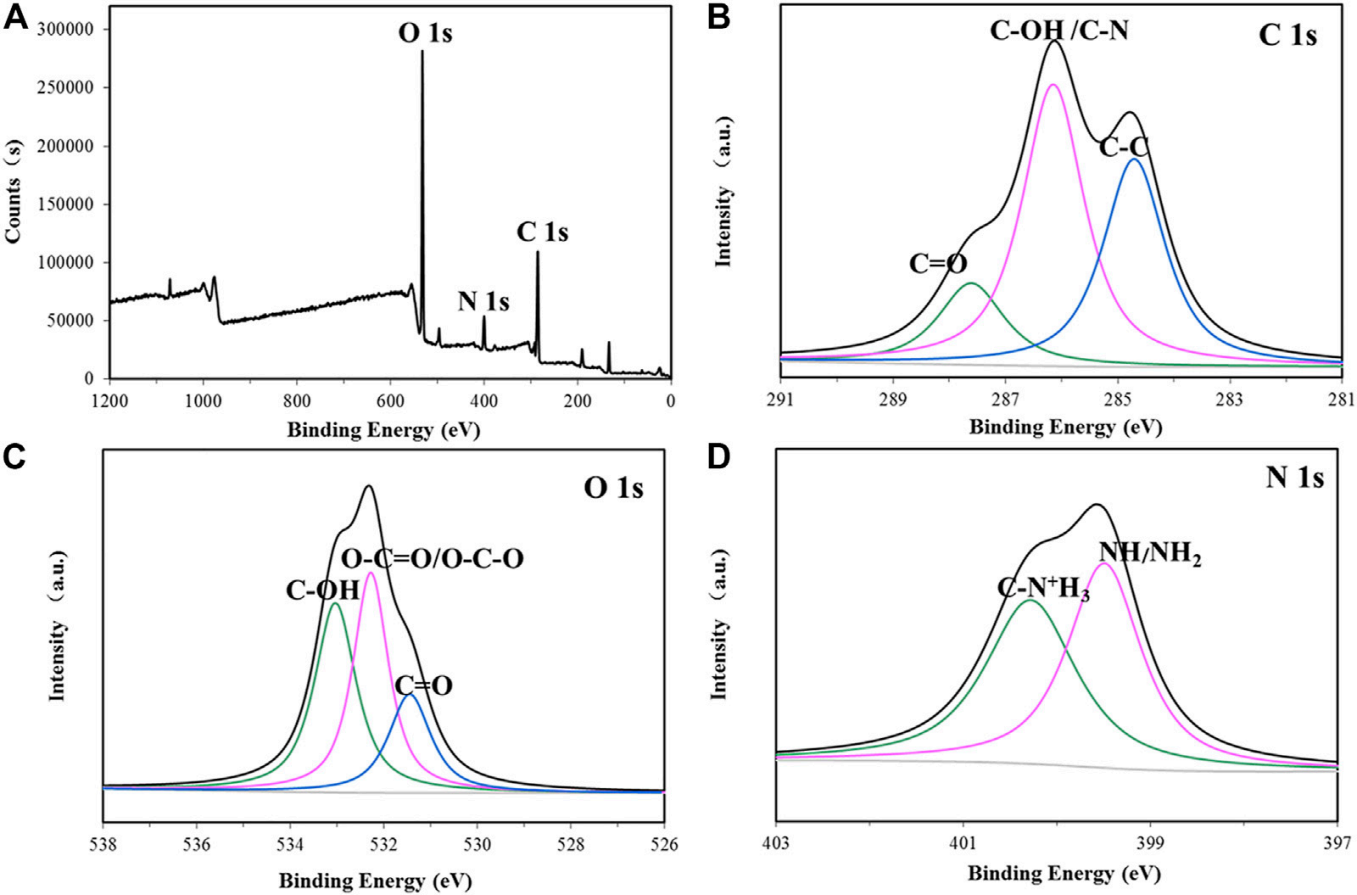
XPS spectra of the bioflocculant **(A)** and high resolution 1 s XPS spectra of C, O, and N from P-GS408 are shown in **(B–D)**, respectively.

The elemental composition of the biofocculant was analyzed by XPS. The mass fraction of C, O, N, S, and P was detected as 63.48%, 30.58%, 4.65%, 1.00%, and 0.29%, respectively. Moreover, the core level peaks of C 1s, O 1s and N 1s were deconvoluted to vertify the corresponding functional groups. As Figure 3 shown, the peaks located at 284.7, 286.2, and 287.6 eV in the C 1s high-resolution spectrum were assigned to C–C, C–OH/C–N and C=O groups, respectively (Li et al., 2009). The peaks located at 531.5, 532.3, and 533.1 eV in the O 1s high-resolution spectrum were attributed to C=O, O–C–O/O-C=O and C-OH, respectively (Liang et al., 2020). The peak at 400.28 eV in the N 1s high-resolution spectrum was assigned to the protonated nitrogen in aminosugar, while the peak at 399.5 eV was originated from the non-protonated nitrogen in NH or NH_2_ group (Zhang et al., 2013; Zhong et al., 2018).

### Measurement of the flocculating activity

**FIGURE 4:**
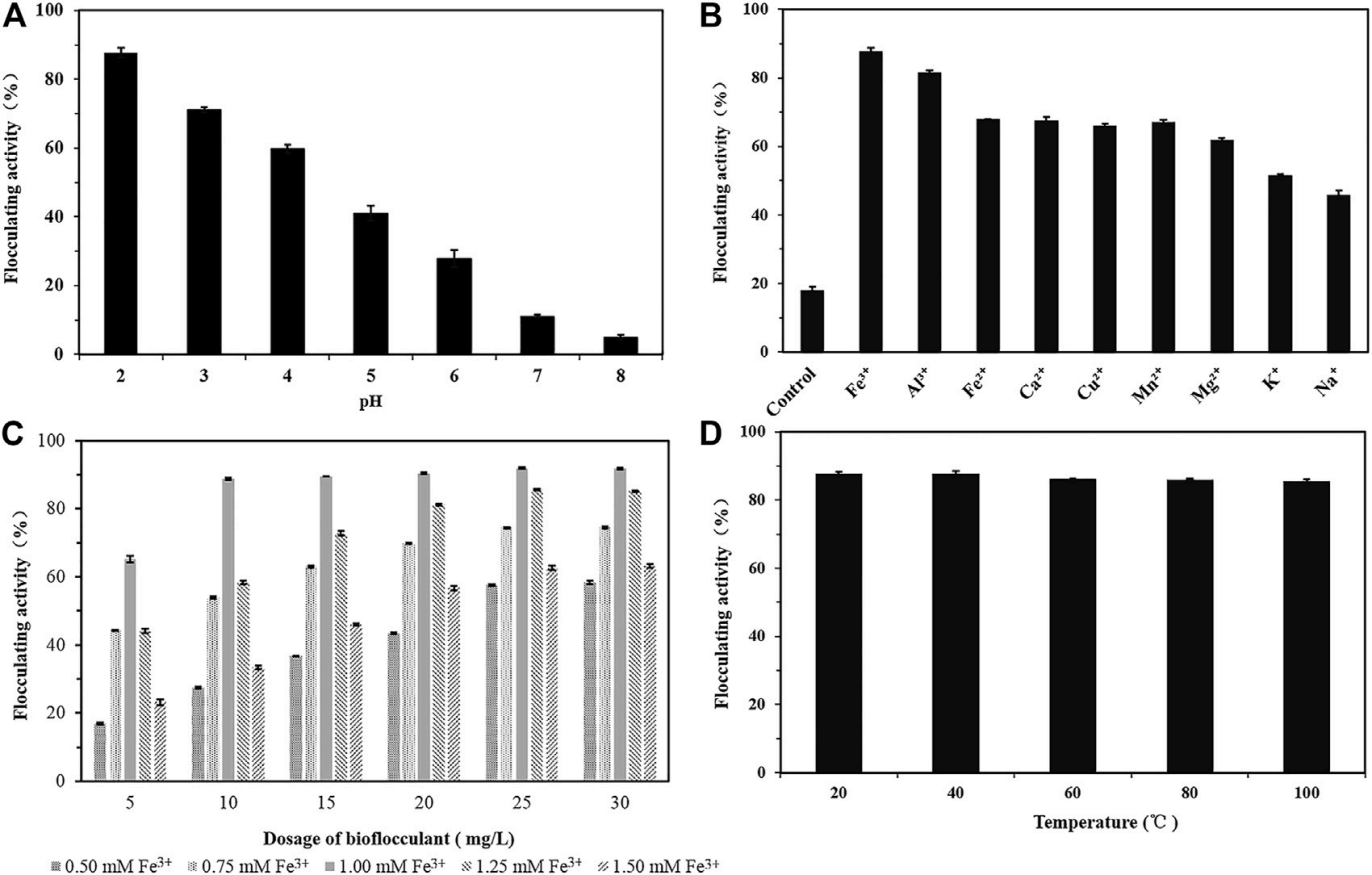
The effects of pH **(A)**, cations **(B)**, dosage of the bioflocculant and Fe^3+^ **(C)**, temperature **(D)** on the flocculating activity of Kaolin suspension by the bioflocculant.

Effects of metal ions, pH, dosage, and temperature on flocculating activity of the bioflocculant were shown in Figure 4. The bioflocculant alone had negligible flocculating activity, but the situation changed by addition of metal ions. At pH 2.0, the bioflocculant at 10 mg/L demonstrated a good flocculating activity of 88.79% and 82.72% toward 4 g/L kaolin suspension, in the presence of 1.0 mM FeCl_3_ and AlCl_3_, respectively. If the metal ion changed or the pH increased, the flocculating activity dramatically dropped. The trivalent cations were more favorable for promoting flocculation than divalent and monovalent cations.

Considering that though the zeta potential of kaolin solution at pH 2.0 was changed from −12.8 to 9.55 mV by addition of the bioflocculant, no obvious fluccolation was observed, however, in the presence of both Fe^3+^ and the bioflocculant, the zeta potential of kaolin solution changed to 35.45 mV and the flocculation efficiency was significantly improved, it was speculated that trivalent cations with higher charge density could more effectively neutralize the negative charge of kaolin particles to form bridges (Salehizadeh et al., 2000). Variations in pH also cause differences in the electrostatic charge of the suspended particles, thus affecting the bridging efficiency for kaolin clay particles. The absolute values of zeta potential of kaolin suspension in the study were increased from −12.8 to −39.9 mV with the increase of pH 2.0 to 6.0.

The synergistic effect of bioflocculant and Fe^3+^ on the flocculating activity was tested at different dosages of bioflocculant and FeCl_3_. The maximum flocculating activity achieved 91.92% at the dosage of 25 mg/L biolfocculant by combination of 1.0 mM Fe^3+^. When the bioflocculant concentration varied from 10 mg/mL to 30 mg/mL, its flocculating activity kept increasing with the increase of Fe^3+^ concentration from 0.5 to 1.0 mM, and at the presence of 1.0 mM Fe^3+^ the flocculating activities were above 88% with not much change during this bioflocculant concentration range, but overdosage of Fe^3+^ would reverse the surface charge of kaolin particles from negative to positive and reduced flocculation obviously by electrostatic repulsion forces (Wang et al., 2015; Nguyen et al., 2021; Hyrycz et al., 2022). With respect to the influence of temperature, the bioflocculant retained high flocculating activity above 85% when temperature varied from 20°C to 100°C, indicated it was stable under high temperature.

### Application in acid mine wastewater treatment

**FIGURE 5:**
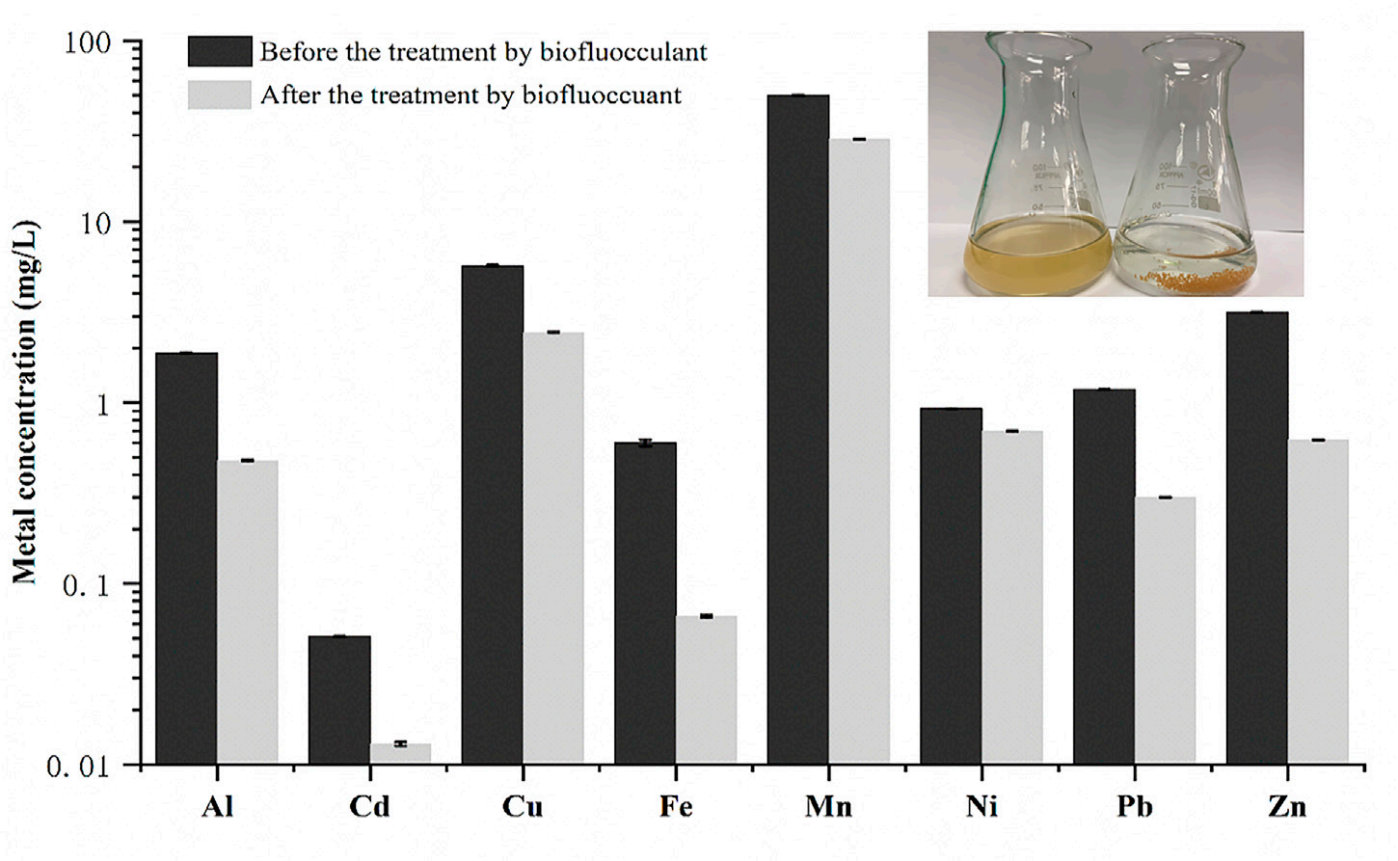
The effect of the bioflocculant on the metal ion concentration of the real acid wine wastewater.

**FIGURE 6:**
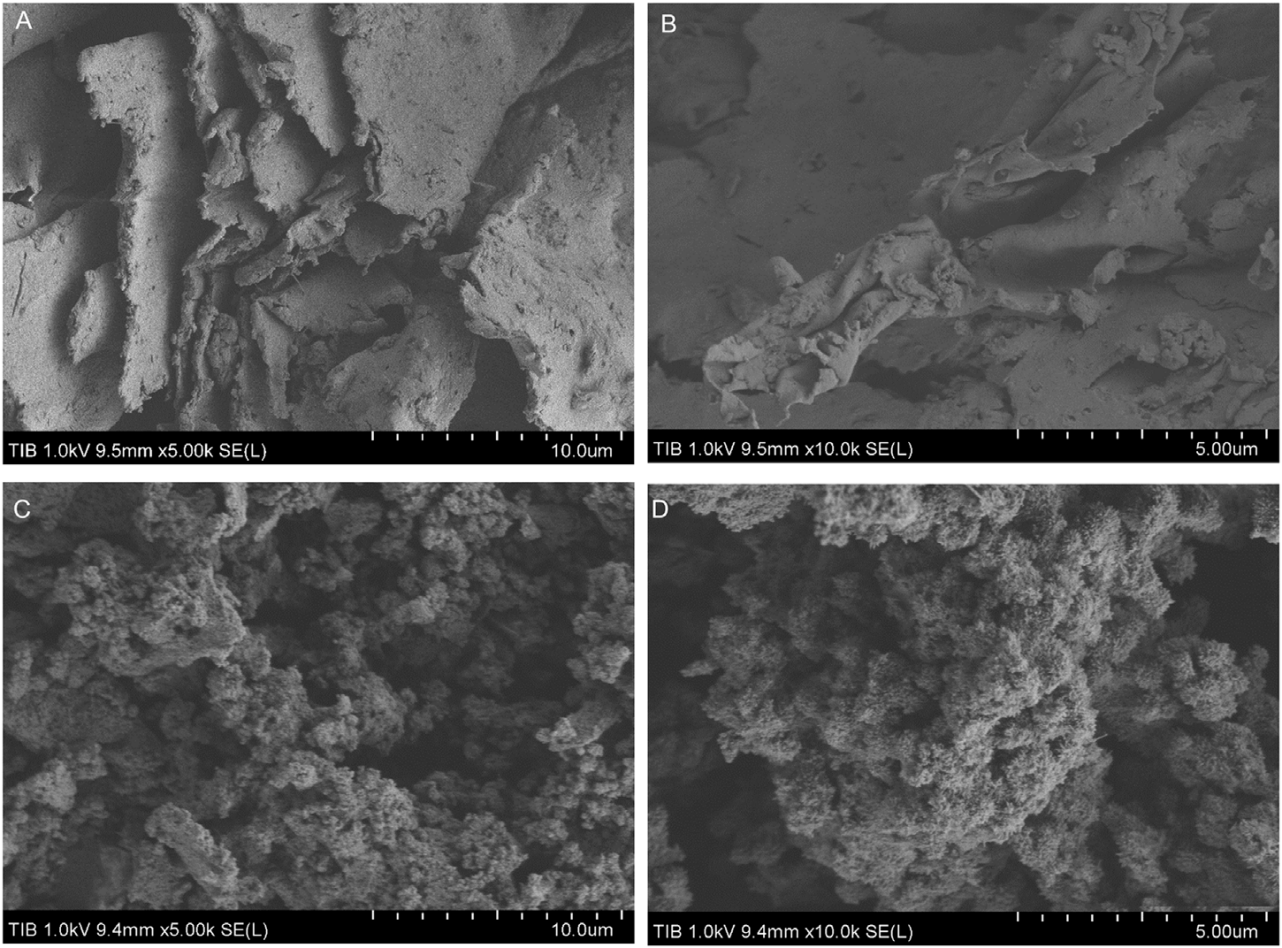
SEM micrograph images of purified bioflocculant **(A,B)** and bioflocculant aggregation with the acid mine wastewater **(C,D)**.

A real acid mine wastewater sample without pH adjustment was directly used to investigate the application possibility of the bioflocculant in treatment of acid mine wastewater. As shown in Figure 5, after treatment with the bioflocculant at a dosage of 25 mg/L for 30 min, the acid mine wastewater sample changed from yellow-brown to colorless and clarified, meanwhile, an amount of yellow-brown sediment appeared at the bottom, and the flocculating activity was tested as 96.39%. The yellow-brown color of the acid mine wastewater was caused by large quantities of ferric ions resulted from the oxidation of ferrous ions, and ferric ions could start to precipitate at a pH as low as 3.0 (Potgieter-Vermaak et al., 2006), it seemed that the addition of the bioflocculant accelerated the precipitation process of ferric ions by flocculation. ICP analysis further confirmed that the bioflocculant was able to remove metal ions from the acid mine wastewater sample, with a removal rate higher than 70% to Fe, Al, Zn, Pb, and Cd. Under the action of the bioflocculant, the final concentration of Pb^2+^ was lower than 1 mg/L, meanwhile, though the final concentration of Mn^2+^ and Cu^2+^ did not meet this standard, their concentration obviously changed from 49.7 to 28.6 mg/L and 5.72 to 2.45 mg/mL, respectively. Moreover, the pH of the acid mine wastewater was effectively increased from 3.04 to 6.86. The zeta potential of the acid mine wastewater was changed from −19.12 to 2.89 mV by addition of the bioflocculant. SEM images showed that the bioflocculant had an irregular stacked layer structure and aggregated with acid mine wastewater to form large clumps with net-like structure (Figure 6).

## Discussion

Fermentation medium accounts for a significant portion in the overall cost of bioflocculant production (Pu et al., 2018; Fan et al., 2019; Mohammed and Dagang, 2019) especially carbon and nitrogen source, plays a vital role (Xia et al., 2018b). Corn steep liquor, the by-product of corn wet-milling, has been used as cheap nutrient supply for the fermentation of various microorganisms, due to its high contents of soluble proteins, amino acids, carbohydrates, vitamins and minerals (de Barros et al., 2020; Kang et al., 2020). The bioflocculant fermentation production process using corn steep liquor as a cheap nutrient supply was successfully accomplished by response surface methodology in this study. According to the *p*-values, the corn steep liquor, glucose and urea all showed significant impact on the yield of bioflocculant production, however, the interaction between glucose, corn steep liquor, and urea was not significant. Though fed-batch cultivation strategy and two-stage pH control mode had been applied to improve the production of bioflocculant REA-11 from *C. glutamicum* CCTCC M201005 (He et al., 2004b; Wu et al., 2010), its reported yield was only calculated in U/mL, thus it was difficult to compare the result with the yield of bioflocculant in this study using the common unit g/L. Meanwhile, considering that only 0.24, 0.26, 0.40, and 0.36 g of purified bioflocculant was obtained from 1 L of fermentation broth of *Klebsiella oxytoca* GS-4-08 (Yu et al., 2016), *Virgibacillus* sp. Rob (Cosa et al., 2011), *Aspergillus flavus* S44-1 (Aljuboori et al., 2013) and *Bacillus amyloliquefaciens* DT (Sun et al., 2015), respectively, the bioflocculant production yield of 0.52 g/L in this study though was not high, but was still acceptable. Besides that *C. glutamicum* was not an efficient bioflocculant producer, it was speculated that the low production yield might be attributed to that only the culture medium composition was partially optimized, other fermentation conditions, such as pH, time and inoculum size, should be optimized in further study.

The monosaccharide composition of the bioflocculant in this study was very different from that of the bioflocculant REA-11 produced by *C. glutamicum* CCTCC M201005, which was composed of galacturonic acid with trifle proteins (He et al., 2002), but had some similarities to many microbial bioflocculants (Sun et al., 2015; Chen et al., 2017; Xia et al., 2018a; Xia et al., 2022). The polysaccharide was the main ingredient in the bioflocculant and the carboxyl groups of uronic acid in the bioflocculant could provide a certain amount of effective sites for the attachment of particles (Xia et al., 2022). The FTIR spectrum showed the presence of carboxyl, hydroxyl, and amino groups in the bioflocculant, and the result of XPS was in agreement with FTIR analysis, further confirmed that the hydroxyl, carboxyl and amino groups were abundant in the bioflocculant (Fan et al., 2019).

The bioflocculant REA-11 originated from *C. glutamicum* CCTCC M201005 was stable at pH 3.0–6.5 and its flocculating activity was markedly improved by Ca^2+^. Under the optimum bioflocculant concentration of 8.2 mg/L and optimum CaCl_2_ concentration of 8.0 mM, its flocculating activity was 85.2% (He et al., 2004a). However, the flocculating activity of the biofloccuant in this study was different from that of REA-11, its enhanced flocculating activity induced by FeCl_3_ or AlCl_3_ was similar to that of the bioflocculants produced by *Bacillus* sp. As-101 (Salehizadeh et al., 2000), *K. oxytoca* GS-4-08 (Yu et al., 2016), *Raoultella ornithinolytica* 160–1 (Ding et al., 2021), *Klebsiellu* sp. A9 (Sheng et al., 2016) and *Halogeometricum borinquense* A52 (Chouchane et al., 2020). Moreover, the maximum flocculating activity of the bioflocculant observed at pH 2.0 was similar to that of the bioflocculants produced by *Bacillus* sp. F19 (Zheng et al., 2008), *Bacillus aryabhattai* PSK1 (Abd El-Salam et al., 2017) and *Azotobacter chroococcum* (Yang et al., 2017). Kaolin clay particles form an electrical double layer at the solid-water interfaces and the negative charge on the surface of the particles enables them to suspend well in the solution. According to Elkady et al. (2011), the good flocculating activity at pH 2.0 might be attributed to that the electric double layer was changed because the negative charge on the surface was reduced by the adsorption of H^+^ in the highly acidic environment, subsequently the electrostatic repulsion force and distance between the suspended particles were decreased, and the bridging effect of the bioflocculant was correspondingly improved. The optimal flocculation conditions of the bioflocculant from *Bacillus* sp. for kaolin solution were determined as 120 mg/L bioflocculant and 0.4 mM Fe^3+^ (Hua et al., 2021). As for the bioflocculant from *K. oxytoca* GS-4-08, the best dosage of the bioflocculant and Fe^3+^ were 700 mg/L and 3.0 mM respectively in the flocculating system (Yu et al., 2016). Compared to these reported bioflocculants, the bioflocculant in this study achieved a high flocculating activity at a relative low dosage by the aid of Fe^3+^. Chouchane et al. (2018) reported that the heteropolysaccharide-based bioflocculant from hydrocarbonoclastic strain *Kocuria rosea* BU22S was thermally stable. Pu et al. (2020) found that the polysaccharide BM2 produced by *Bacillus megaterium* strain PL8 had a flocculating activity over 87% from 20°C to 100°C. The thermostability of the bioflocculant in this study is in accordance with these reports. Moreover, considering that biofluccolants with polysaccharide backbone were thermostable, while those with high compositions of proteins were generally sensitive to heat (Tawila et al., 2018), the good thermo-stability of the bioflocculant indicated that its flocculating activity was primarily resulted from the polysaccharide-based structure, rather than protein.

Acid mine wastewater has a low pH of 2.0–3.0 and contains high levels of different metals such as iron, aluminium, manganese, zinc, lead, copper and nickel (Vital et al., 2018). The high acid value and the existence of the metal ions in acid mine wastewater are both beneficial to the performance of the bioflocculant in this study. The flocculation behavior of the bioflocculant in the real acid mine wastewater sample illustrated that some metal ions underwent co-precipitation with ferric ions and was removed from the wastewater by the help of the bioflocculant. Meanwhile, due to the precipitation of sulfides, hydroxides, and carbonate (Chen et al., 2021), the pH of the wastewater changed from acid to close to neutral. After treatment by the bioflocculant, the final concentration of Pb^2+^ and the pH in the wastewater sample reached the national wastewater standard stipulated in the Integrated Wastewater Discharge Standard (Yan et al., 2015). The zeta potential change of the acid mine wastewater with the addition of the bioflocculant showed that the charge neutralization mechanism was involved in the flocculation. Moreover, the interstitial spaces between the stacked layers of the bioflocculant observed by SEM were beneficial to the absorption of metal ions (Saba et al., 2019), while the net-like structure of the flocs after flocculation indicated that polymer bridging played an important role in the flocculation process (Aljuboori et al., 2015). Considering that the mineral content and concentration of water-soluble metal ions in the acid mine water varies with the geological environment in different mining areas, in the further study, the action of the bioflocculant needs to be tested using more different acid mine water samples, at the same time, its application condition requires more detailed exploration.

In conclusion, a novel cation-dependent bioflocculant was produced by the GRAS strain *C. glutamicum* utilizing corn steep liquor as a cheap nutrient supply. Chemical analysis indicated that the bioflocculant was mainly composed of polysaccharides (53.85%) and proteins (16.51%). FTIR and XPS analysis revealed the abundant carboxyl, amine and hydroxyl groups in its structure. The bioflocculant had high thermal stability, and showed a good flocculating activity over 70% to kaolin clay suspension by the aid of Fe^3+^ at high acidic condition. Moreover, the simple addition of bioflocculant to the real acid mine wastewater could remove metal ions such as Fe, Al, Pb, Zn efficiently from the sample, making the suspension clear with an increase in the pH to neutral. These findings indicated the bioflocculant from *C. glutamicum* could be an efficient and promising material to treat acid mine wastewater in a simple and fast way.

## Data Availability

The raw data supporting the conclusions of this article will be made available by the authors, without undue reservation.

## Supplementary material

Supplementary Figure S1 The 16S rRNA gene sequence of *C. glutamicum* Cg1-P30.

Supplementary Figure S2 Neighborhood joining tree of *C. glutamicum* Cg1-P30.

Supplementary Figure S3 SEM micrograph images of purified bioflocculant (A), Kaolin (B) and bioflocculant aggregation with Kaolin (C).
