# Supplementary figures and images for: A bioflocculant from Corynebacterium glutamicum and its application in acid mine wastewater treatment
Source: Front Bioeng Biotechnol. 2023 Feb 28;11:1136473. doi: 10.3389/fbioe.2023.1136473 (PMC10011464; doi:10.3389/fbioe.2023.1136473)

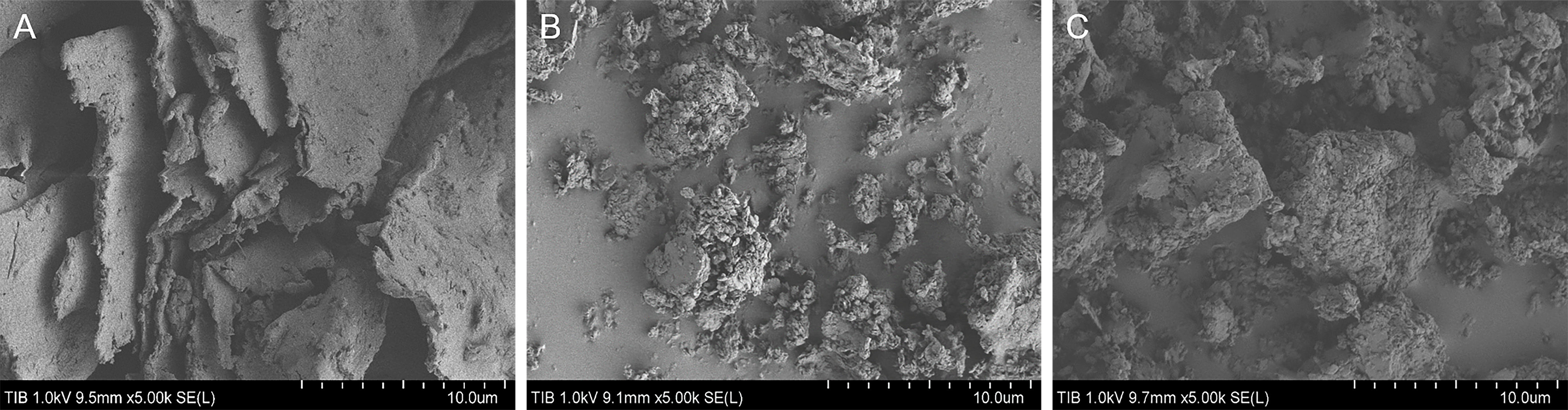

Supplement: Supplementary file 1 [file Image3.TIF]

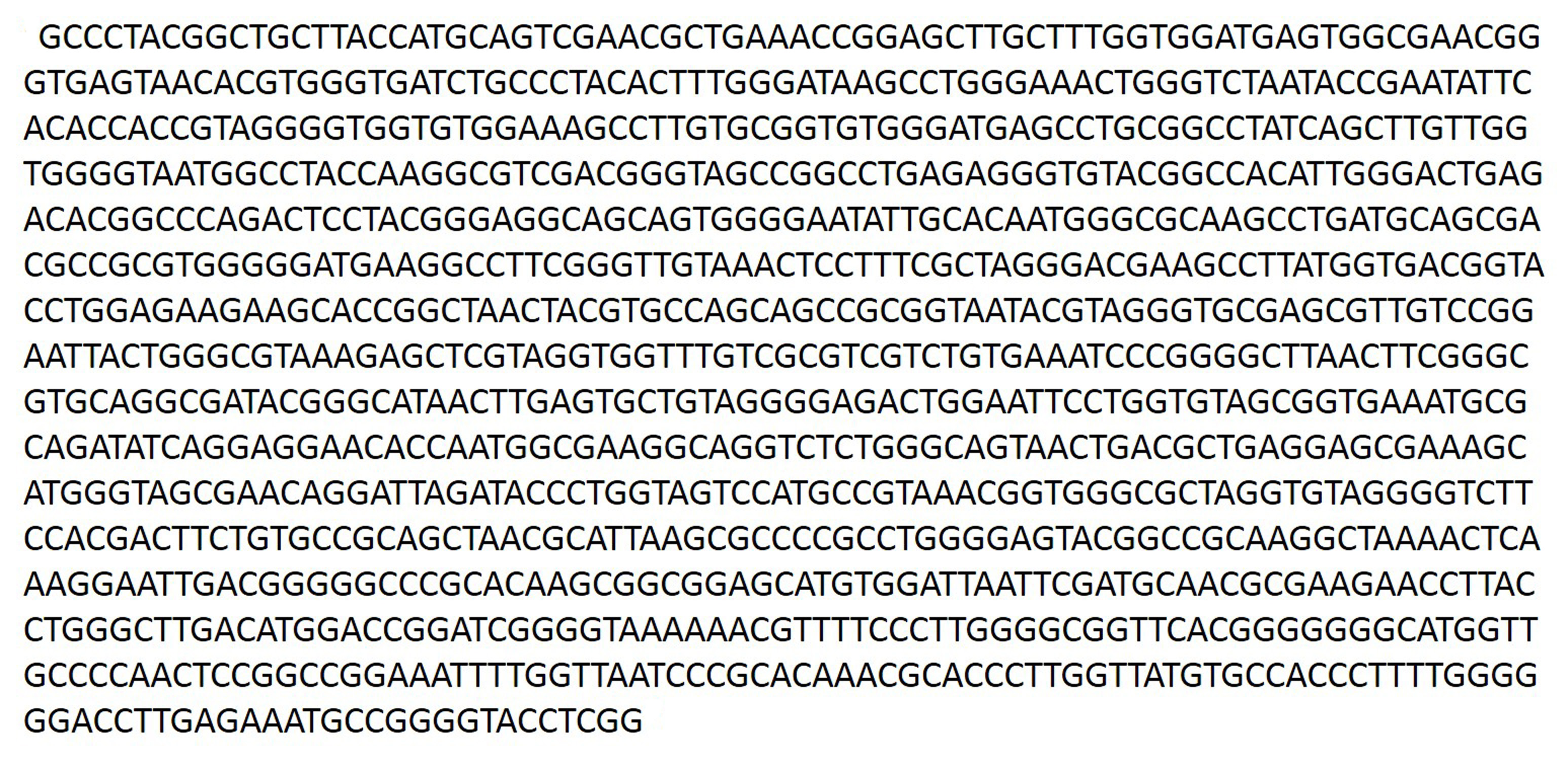

Supplement: Supplementary file 2 [file Image2.TIF]

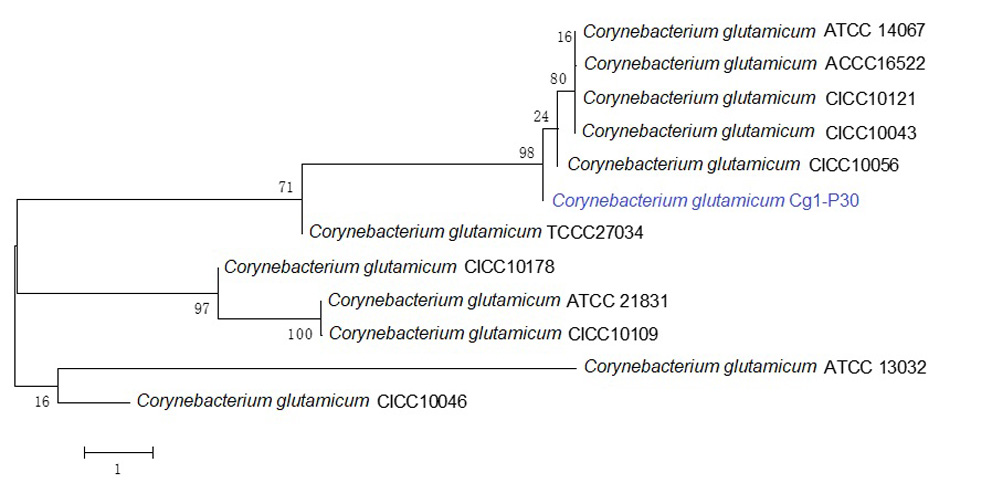

Supplement: Supplementary file 3 [file Image1.TIF]
